# Immune Microenvironment in Oral Potentially Malignant Disorders and Oral Cancer: A Narrative Review

**DOI:** 10.3390/ijms26146650

**Published:** 2025-07-11

**Authors:** Aiman Ali, Graziella Rigueira Molska, Huiling Yeo, Najmeh Esfandiari, Will Jeong, Michelle Huang, Marco Magalhaes

**Affiliations:** 1 Faculty of Dentistry, University of Toronto, Toronto, ON M5G1G6, Canada; aiman.ali@utoronto.ca (A.A.); grazi.rigueiramolska@utoronto.ca (G.R.M.); huiling.yeo@gmail.com (H.Y.); tina.esfandiari@utoronto.ca (N.E.); yuchen.huang@mail.utoronto.ca (M.H.); 2 Schulich School of Medicine & Dentistry, Western University, London, ON N6A5C1, Canada; wjeong2027@meds.uwo.ca; 3 Dental and Maxillofacial Sciences, Sunnybrook Health Sciences Centre, Toronto, ON M4N3M5, Canada

**Keywords:** inflammation, immune cells, OSCC, OPMDs, tumor immune microenvironment

## Abstract

Multiple studies have investigated the impact of the tumor immune microenvironment (TIME) on oral squamous cell carcinoma (OSCC), with most focusing on three key cellular components: lymphocytes, macrophages, and neutrophils, as well as the molecular mechanisms underlying inflammation-mediated OSCC invasion. Although the specific roles of each cell type vary depending on their subtypes and the characteristics of OSCC, several consistent patterns have been identified. TIME plays a critical role at every stage of OSCC progression, from tumor initiation and growth to invasion and metastasis. Understanding the communication signals–the language–between tumor cells and the TIME, encoded through various proteins secreted by immune cells, is essential for controlling tumor progression and developing effective treatments for OSCC. This review provides an overview of how TIME influences the progression of the Oral Potentially Malignant Disorders (OPMDs) to OSCC as well as OSCC’s invasion, focusing on the contributions of various immune cells within the TIME. Additionally, we discuss recent advances in immunotherapy for OSCC, highlighting strategies to enhance immune responses and improve treatment outcomes.

## 1. Oral Cancer and Oral Potentially Malignant Disorders (OPMDs)

Oral and pharyngeal cancer are some of the most common cancers in the world, ranking as the sixth most common cancer [1] with more than 400,000 new cases annually [2].

About 94% of oral cancers occur in older people above the age of 45 years [3]. Only a few cases of oral cancer may affect young patients.

Oral cancer has a multifactorial etiology. Smoking, chewing tobacco and betel nut, snuff dipping, and alcohol misuse are established risk factors for oral cancer. Sunlight is a strongly suggestive risk factor for lip cancer, while other possible risk factors for oral cancer may include viruses, radiation, and immune deficiency [1]. Smokers and heavy drinkers have a 38 times higher risk of developing oral and pharyngeal cancer than nonsmokers and non-drinkers [4]. The use of topical steroids was reported recently as an additional important risk factor associated with the malignant transformation of OPMDs to OSCCs [5]. On the opposite side, a diet rich with fruit and vegetables was found to reduce the risk of developing oral cancer by at least a quarter [6]. This suggests that a diet deficient in antioxidants is a predisposing factor towards the development of oral cancer [7,8] as well as for potentially malignant disorders [9].

**Figure 1:**
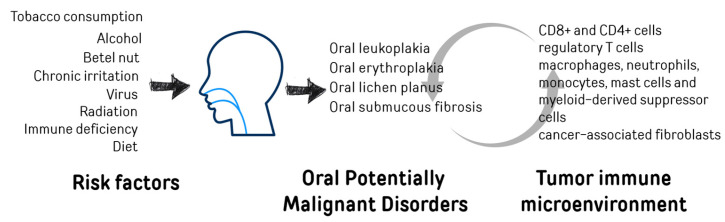
Risk factors related to the progression from normal oral mucosa to OPMDs and OSCCs.

It was documented that oral cancer risk factors, mainly alcohol consumption and smoking, can strongly influence tumor growth by negatively impacting the tumor immune microenvironment (TIME) [10,11] (Figure 1).

The 5-year survival rate for a localized oral cancer tumor is greater than 80%. This survival rate usually drops to 50% in cases of regional metastasis, and it drops to less than 30% in cases of distant metastasis [12]. Oral cancer screening programs and the early detection of OPMDs and oral cancer dramatically improve the prognosis and reduce mortality from oral cancer [13]. Similarly, the association of OSCC with precursor lesions showed a significant improvement of the prognosis with a lower mortality compared to OSCCs without association with precursor lesions [14].

Oral potentially malignant disorders (OPMDs) are those lesions that have an increased risk of transformation into oral cancer. OPMDs may appear clinically as white, red, and mixed white and red lesions, and they may also appear as plaque/plateau, smooth, corrugated, granular, verrucous, or atrophic lesions with variable size [15,16].

These disorders include Leukoplakia, Proliferative Verrucous Leukoplakia (PVL), Erythroplakia, Oral Submucous Fibrosis (OSF), Oral lichen planus (OLP), Actinic Keratosis (Actinic Cheilitis), Palatal Lesions in Reverse Smokers, Oral Lupus Erythematosus (OLE), and Dyskeratosis Congenita (DC) [17].

The ratio of progression of OPMDs to cancer varies from 1.4% to 49.5% throughout a period ranging from 12 months to 20 years [18]. Squamous cell carcinoma is the most common cancer arising in patients with OPMD.

The first step of the malignant transformation of the OPMDs may occur when the OPMDs’ epithelial cells start communicating with the surrounding immune microenvironment by shaping it and making the TIME ready for seeding the tumor cells. Recently, it was shown that OPMDs can promote carcinogenesis by remodeling the adjacent microenvironment and making it a favorable condition for the initiation of oral cancer [19]. This may reflect the importance of early detection of OPMDs and treating them as the first line of defense in controlling oral cancer [20].

## 2. Tumor Immune Microenvironment–TIME

**Figure 2:**
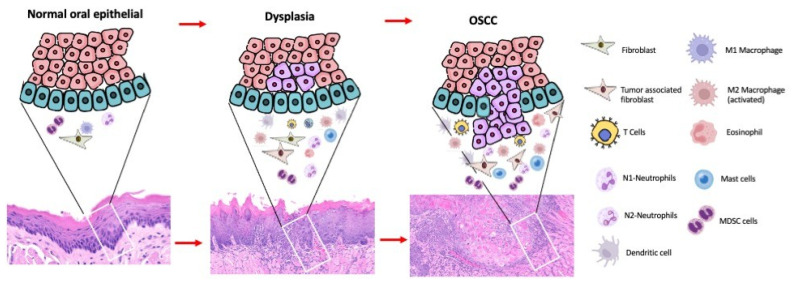
Changes in the TIME through the progression from normal epithelium, dysplastic epithelium to OSCC.

The tumor immune microenvironment is a complex ecosystem that includes diverse cell types, such as adaptative immune cells (e.g., CD8+ and CD4+ cells, regulatory T cells, among others) [21] and myeloid immune cells (e.g., macrophages, neutrophils, monocytes, mast cells, and myeloid-derived suppressor cells, among others) [22].There are also cancer-associated fibroblasts promoting immunomodulatory activities contributing to immune evasion, the remodel of the extracellular matrix, and tumor progression [23]; as well, the blood vessel endothelial cells provide plasticity, controlling the passage of oxygen, cells, and fluids [24]. Many other markers and changes in the TIME were reported to favor the malignant transformation of OPMDs or even the metastasis of OSCCs, those included: PD-1 PDL-1 [25], MMPs Cortactin FISH and invadopodium [26], TNFα [27], and salivary immune cells and mediators [28]. TIME may differ according to the intrinsic features of cancer cells, patients, and cellular composition which can promote the suppression or progression of the tumor [29] (Figure 2).

## 3. Tumor Associated Myeloid-Derived Suppressor Cells (MDSCs) and Dendritic Cells (DCs)

MDSCs exert suppressive activity on NK, T, and B cells leading to tumor immune evasion. Recent studies have explored the TIME and crosstalk mechanism of DCs and MDSCs contributing to SCC malignant progression [30,31]. Tumor-related MDSCs enhance OSCC progression through cell proliferation, migration, and invasion leading to the poor prognosis of patients. Additionally, OSCC cells can induce MDSCs differentiation by upregulating the expression of arginase-1 (Arg-1) and inducible nitric oxide synthase (iNOS) [30]. Clinical data from patients, including biopsies and blood samples, have demonstrated the infiltration of DCs and MDSCs in an OSCC malignant tumor, some of which include lymph node metastasis or premalignant OPMDs. These cells were activated via the TNF-α/NF-κB/CXCR-4 pathway. Furthermore, monocytes-derived MDSCs (M-MDSCs) express higher levels of phosphorated STAT3 (pSTAT3), interleukin-10 (IL-10), and programmed death-ligand 1 (PD-L1) compared to polymorphonuclear MDSCs (PMN-MDSCs). M-MDSCs are effective in suppressing the T cells’ proliferation by inhibiting interferon-gamma (IFN-γ) production and promoting regulatory T cell (Treg) generation via the transforming growth factor beta (TGF-β). However, M-MDSCs also promote the Th17 cell through cyclooxygenase 2 (COX-2) and oxide synthase (NOS). The interplay between MDSCs and DCs appears to play a crucial role in regulating epithelial cells’ behavior and promoting tumor progression [31,32,33]. Cytometry analysis of tumor cell samples from the lymph nodes of OSCC patients revealed high PD-L1 expression in dendritic cells (DCs). This was associated with reduced T cell efficacy, contributing to a poor prognosis [34].

In addition, experimental studies, using mouse models provide valuable insights into the role of TIME and serve as guides for developing future therapeutic combination approaches in OSCC. TIME analysis in mouse tumor models has revealed an increase in CD155 + PD-L1 + T cells and effector memory MDSCs during the middle and late stages of tumor progression. A blockade of TIGIT/CD155 and PD-1/PD-L1 signaling pathways in these models significantly inhibited tumor growth, enhanced the proportions of effector T cells, and increased cytokine secretion [35].

The use of small molecules and inhibitors further enhances the understanding of the MDSCs’ role in the OSCC. For instance, in OSCC models induced by chemical carcinogens (mimicking tobacco-associated OSCC) or MOC2 injection, inhibitors targeting PI3K (associated with granulocytes) and CXCR1/2 (i.e., a myeloid chemokine receptor inhibitor) effectively reduced tumor evasiveness and burden [36]. Moreover, SX-682-mediated MDSC inhibition has been shown to enhance the efficacy of NK cell transfer therapy [37].

In a mouse xenograft model, the CCL2/CCR2 axis was found to play a critical role in mediating crosstalk between cancer cell and stromal components within the TIME, underscoring its significance in OCSS progression [38].

Inflammatory states can lead to cancer cell proliferation and invasion. Combining a high-fat diet (HFD) and oral carcinogenesis induced by 4NQO, Peng J et al., 2021, showed an increase in invasive OSCC compared to regular-diet non-obese mice and changes in the TIME, promoting an expansion of the MDSCs recruited through the CCL9/CCR1 pathway but only for HFD mice. In addition, the tumor tissue of obese patients showed a positive correlation between an increase in MDSCs and local adipocytes in OSCC [39]. Even though the infiltration of CD33+ MDSCs is significantly higher in OSCC than in OPMDs, MDSCs play an important role in the carcinogenesis of OPMDs, as opposed to DCs, which are proven to inhibit the carcinogenesis of OPMDs [19].

## 4. Tumor-Associated Mast Cells

Mast cells (MCs) infiltrate the TIME, exhibiting both anti-tumor and pro-tumor activities depending on the type of stimulus, and they play a regulatory role in tumor progression across various cancer types. They regulate tumor progression in different tumor types. Single-cell RNA sequencing and fluorescence in situ hybridization were used to develop an MC-associated risk signature model based on immune infiltration in tissue sections from patients with HNSCC. This model identified nine MC-related prognostic genes: KIT, RAB32, CATSPER1, SMYD3, LINC00996, SOCS1, AP2M1, LAT, and HSP90B1. High expressions of RAB32, CATSPER1, SMYD3, AP2M1, and HSP90B1 were associated with a poor prognosis, while high levels of the other genes were correlated with an improved prognosis. This model could serve as a tool for patients’ stratification and the identification of immunotherapeutic strategies [40].

The influence of MCs on OSCC progression is well-documented, although the molecular mediators underlying this relationship have only recently been elucidated. MCs can modulate tumor progression either directly or indirectly, depending on their localization within the tumor or at a distance from the TIME. This interaction can result in contrasting effects, such as reduced tumor cell invasion or enhanced tumor cell proliferation. For instance, co-culture with CC chemokine ligand 2 (CCL2) has been shown to increase tumor proliferation. Conversely, indirect stimulation by MCs has been reported to reduce tumor cell invasion [41].

The mast cells were also shown to be involved in the OPMDs’ carcinogenesis through promoting angiogenesis, and like MDSCs, their density statistically increases when moving from normal tissue to OPMDs and then OSSCs [42,43].

## 5. Tumor-Infiltrating Lymphocytes (TILs)

The influence of TILs on OSCC is consistent, with several studies agreeing that CD8+, CD45RO+, CD57+ TILs, and CD4+ central memory T cells (CD45RO+/CCR7+) are all correlated with improved prognosis. As in other tumors, CD8+ cytotoxic T lymphocytes (CTL) are thought to improve survival by directly killing malignant cells expressing tumor-associated peptides on class I major histocompatibility molecules (MHC I). Given these roles, certain studies have aimed to understand TIL recruitment and OSCC defenses. The knockdown of CXCL14 in OSCC lines (MOC1 and MOC2) injected into immunocompetent mice slows tumor growth in a TIL-dependent fashion, suggesting that CXCL14 may have a protective role. Furthermore, metastatic OSCCs express less CXCL14 than the primary tumors, supporting the notion that CXCL14 are implicated in TIL recruitment and tumor suppression [44].

In contrast, the transforming growth factor-β (TGF-β) is elevated in most OSCCs as a product of the tumor or the surrounding TIME. As a well-established immunosuppressive cytokine, TGF-β can inhibit the cytotoxic capabilities of CD8+ cells. Inhibition of TGF-β in a CTL OSCC co-culture conversely improves cytotoxicity and reduces OSCC growth. As such, the direct or indirect control of TGF-β expression may be a primary mechanism by which OSCCs suppress CTL function [45].

However, directly improving the function of CD8 or CD4 TILs to combat tumor growth may prove premature. Specifically, regulatory T cell ablation in 4-NQO-induced OSCC in murine models upregulates the effector T cell activity/proliferation but also promotes OSCC incidence and burden. This increase in malignancy is likely TIL-dependent, as the subsequent depletion of CD4 and CD8 antibodies abrogates these consequences [46].

The role of T lymphocytes in the TIME of OPMDs is not clear yet. While some studies documented more CD4+ and CD8+ T cell infiltration in OSCCs compared to OPMDs, others showed more T cell infiltration in OPMDS [47,48].

## 6. Tumor-Associated Macrophages

Tumor-associated macrophages (TAMs) are the predominant leukocytic component of the OSCC TIME, primarily residing in the tumor-adjacent stroma rather than OSCC nests. The majority of TAMs are monocyte-derived, though tissue-resident macrophages have also been identified [49]. These monocyte-derived macrophages are attracted by several chemotactic cues produced by the tumor, including CCL2/MCP1 (monocyte chemotactic protein-1), CCL3/MIP-1α (macrophage inflammatory protein-1alpha), CCL4/MIP-1β (macrophage inflammatory protein-1beta), and CCL5/RANTES (regulated on activation, normal T cell expression and secretion).

While the literature on TILs is fairly consistent, the influence of macrophages on OSCC is somewhat less unclear. TAMs have been shown to either promote or inhibit OSCC proliferation, invasion, and migration under different circumstances. These contrasting functions have been explained through M1 and M2 models of macrophages, which suggest that macrophages can be polarized to an anti-tumor M1 (classically activated macrophages) or pro-tumor M2 (alternatively activated macrophages) phenotype.

M1 polarization is thought to be induced by bacterial PAMPs or interferon gamma (IFN-γ) from Th1 cells. The primary way M1 cells are thought to be anti-tumor is through their improved antigen presentation capabilities, which are the result of higher MHC II expression and lysosomal activity. This improves CD4+ T cell activation, triggering tumor-specific CTL expansion and limiting OSCC growth. Patient data supports this, as M1-polarized TAM abundance is strongly correlated with CTL activation. M1 cells also produce ROS/RNS, leading to direct cancer cytotoxicity, as well as proinflammatory cytokines, including IL-12, IL-23, and TNFα, to recruit neighboring immune cells.

In contrast, Th2-derived anti-inflammatory cytokines such as IL-4, IL-10, and IL-13 are thought to polarize M2 macrophages. OSCC TAMs are primarily M2, and several pathways by which OSCCs promote this polarization have been identified. For instance, OSCCs from the Cal-27 and SCC-9 lines overexpress the miR-29a-3p. This microRNA is then encapsulated in exosomes and capable of suppressing macrophage SOCS1 expression, activating STAT6 signaling, which promotes M2 polarization [50]. Furthermore, Cal-27 and SCC25 release exosomes with CMTM6 on the surface that can activate the ERK1/2 signaling in macrophages to induce M2 polarization [50]. OSCCs have also been determined to overexpress CCR7 to promote macrophage recruitment and M2 polarization, though the precise mechanism is still unclear [51]. Once induced, M2 cells produce a wide range of immunosuppressive effects including Foxp3^+^Treg induction and the production of soluble cytokines/receptors including the IL-1 receptor antagonist, IL-10, and TGF-β. M2 cells also express abundant surface PD-L1 and secrete Arg1 to inhibit T cell development. This combined immunosuppressive environment indirectly aids OSCC growth. M2 cells have also been recognized to be capable of directly facilitating OSCC growth. For instance, TGF-β triggers the TβRII/Samd3 axis in OSCCs, inducing VEGF expression. TAMs can also directly produce other growth factors (ex. epidermal growth factor, fibroblast growth factor, and platelet-derived growth) in addition to matrix metalloproteinases (MMPs), promoting invasion. Consistent with these results, the reconstitution of xenograft OSCC models with M2 macrophages promotes tumor progression, vascularization, and recurrence after therapy.

However, some evidence suggests that M1 macrophages may in fact promote tumor growth and invasion [52]. For instance, exosomes from Cal-27 and SCC25, the same cell lines suggested by Tang et al. to induce M2 polarization [50], were later suggested by You et al. to trigger M1 polarization instead [53]. These M1 cells released IL6, increasing OSCC malignancy and epithelial–mesenchymal transition markers. IL6 could also stimulate the Jak/Stat3 axis, promoting the formation of exosomes with THSB1 that could trigger more M1 polarization. In SCC25, conditioned media from M1 macrophages have been shown to improve OSCC development in vitro and in vivo by triggering the ErbB2/phosphoinositide 3-kinase (PI3K)/AKT/ERK pathway. Another possible explanation for the observed pro-tumor role of M1 macrophages is their ability to produce TNF-α, which several studies have identified to be a strong promoter of OSCC growth and invasion.

With regard to the macrophages’ infiltration and the TIME of OPMDs, it was found that CD163^+^M2 macrophages are positively associated with the carcinogenesis of OPMDs [54]. Moreover, CD163^+^M2 macrophage infiltration increases with higher degrees of dysplastic OPMDs [55].

## 7. Tumor-Associated Neutrophils (TANs)

More recently, interest in the role of tumor-associated neutrophils (TANs) on OSCCs has grown. Neutrophil infiltration in OSCC is generally associated with poor clinical outcomes; accordingly, a neutrophil to lymphocyte ratio (NLR) has been identified as a reliable prognostic marker for shorter overall survival [56]. In most cancers, neutrophil recruitment to the TIME is mediated by several inflammatory cytokines including TNFα, IFNγ, GM-CSF, and IL-8. OSCCs can exploit these pathways to trigger neutrophil recruitment in several ways. For instance, VAP1 overexpression in OSCCs can act in an autocrine manner to trigger NFκB, eventually leading to IL-8 secretion [57].

Once present, TANs are hypothesized to be polarized to either an N1 or N2 phenotype, like that of macrophages. This polarization is dependent on the cytokines present at the local microenvironment, with IFN-β inducing the N1 phenotype while TGF-β induces the N2 phenotype. N1 TANs are typically considered to have an anti-tumor effect, activating both the innate and adaptive system. Notably, these TANs are an effective APC which express co-stimulatory molecules upon stimulation with GM-CSF or IFN-γ. They also facilitate cytotoxicity directly (through the production of apoptotic mediators and ROS) and indirectly via antibody-dependent cellular cytotoxicity (ADCC). In contrast, N2 TANs are generally pro-tumor and can promote tumor growth through elastase secretion while releasing MMP8, MMP9, and VEGF to facilitate metastasis and angiogenesis. Furthermore, N2 TANs can secrete arginase, suppressing T cell activation, and release IL-18, inhibiting NK cell activation [58].

As the OSCC TIME is elevated in IL-1β, IL-6, and TGF-β, it is thought that N2 neutrophils play the predominant role in OSCC development. However, as with M1 TAMs, N1 TANs may have a larger role than conventionally established. Co-culturing neutrophils and OSCC increases TNFα secretion from neutrophils, a hallmark of N1 TANs. TNFα stimulation can induce MMP secretion and invadopodia formation in a PI3K-dependent manner, upregulating invasion. With TNFα expression in the lamina propria also higher in patients with progressing OSCCs, neutrophils may in fact promote tumor formation under certain conditions [59].

In OPMDs, the conversion from the N1 to N2 phenotype of neutrophils may favor the carcinogenesis progression [60]. Also, neutrophils may affect the OPMDs’ TIME and progression through the production of neutrophil extracellular traps (NETs) [61].

On the other hand, the exact role of tumor-associated tissue eosinophilia and oral cancer development is still questionable. While some studies suggested that tumor-associated tissue eosinophilia increases in OPMDs and OSCCs; however, other studies showed negative prognostic outcomes [62].

## 8. Cancer Associated Fibroblast (CAF)

Fibroblasts are integral components of the tumor immune-microenvironment (TIME) and exhibit a complex duality in their functional roles that can significantly influence tumor progression. Numerous studies have demonstrated that fibroblasts can exert tumor-suppressive effects through various mechanisms. Conversely, other research indicates that fibroblasts can undergo a transformation into cancer-associated fibroblasts (CAFs) in response to signals from the tumor. In this activated state, CAFs can promote tumor growth. This dynamic shift in fibroblast identity underscores their complex role in cancer biology, highlighting the importance of understanding their behavior in the TIME for developing effective therapeutic strategies [63].

Fibroblasts represent one of the most abundant cell types within the stroma. These cells are instrumental in the synthesis and remodeling of various extracellular matrix (ECM) components, which are essential for the maintenance of tissue homeostasis and functionality [64]. They contribute to the production of several extracellular matrix (ECM) proteins, including fibrillar collagens (types I, III, and V), proteoglycans, fibronectin, glycosaminoglycans, and various other glycoproteins and fibrillar elements. Collectively, these substances establish a three-dimensional framework that generates osmotic-active scaffolds within the stromal interstitial tissue [64,65].

Cancer-associated fibroblasts (CAFs) can originate from a variety of sources, including local fibroblasts, endothelial cells, hematopoietic stem cells, preadipocytes, and tumor epithelial cells through the process of epithelial–mesenchymal transition. These cancer-associated fibroblasts (CAFs) act as “factories” that generate a diverse array of proteins, signaling molecules, cytokines, and growth factors. They enhance tumor angiogenesis and promote tumor invasion, thereby contributing to tumor progression and metastasis [66,67]. They can be identified both in vitro and in vivo through a panel of marker proteins/genes such as PDGFR, SMA, fibroblast-associated protein (FAP), and fibroblast-specific protein 1 (FSP1) [68].

Cancer-associated fibroblasts (CAFs) are a key component of the tumor stroma in many cases of oral squamous cell carcinoma (OSCC) [69]. Their presence is associated with resistance to immunotherapy and correlates with poorer disease-free survival outcomes in patients [70,71]. Additionally, CAFs have been detected in potentially malignant lesions as well as in histologically normal mucosa adjacent to OSCC (HNMOSCC). This indicates that CAFs may influence the tumor microenvironment and contribute to the progression of disease, suggesting their potential role in early tumorigenic changes [72,73].

Immunohistochemical staining of the alpha-smooth muscle actin (αSMA) was utilized as a marker for cancer-associated fibroblasts (CAFs), and Kellerman’s scoring criteria were applied to evaluate their frequency in oral squamous cell carcinoma (OSCC). Datar et al. demonstrated that the frequency scores of αSMA-positive CAFs in OSCC ranged from 1 to 4. Notably, approximately 40% of OSCC cases exhibited a CAF score of three, indicating that 41–60% of the stromal area was occupied by CAFs. In contrast, among cases of histologically normal mucosa adjacent to oral squamous cell carcinoma (HNMOSCC), 13.3% showed no expression of CAFs, while the remaining cases exhibited a CAF score of one. The presence of cancer-associated fibroblasts (CAFs) in histologically normal mucosa adjacent to oral squamous cell carcinoma supports the hypothesis that CAFs contribute to field cancerization and may facilitate the development of second primary tumors [74].

**Figure 3:**
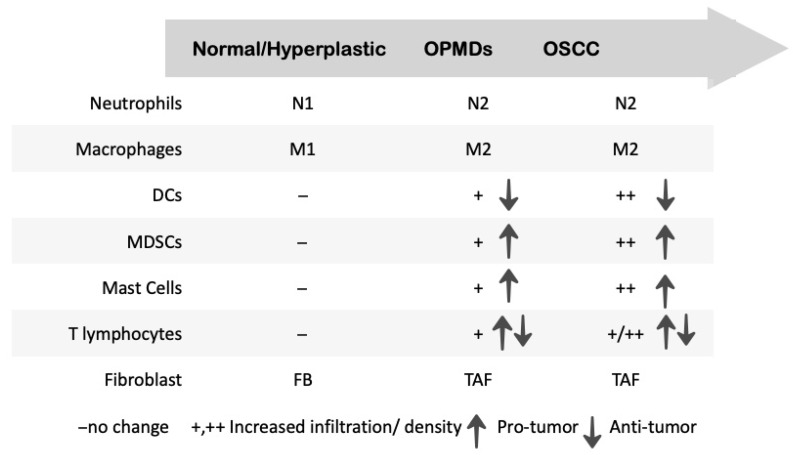
Tumor immunomicroenvironment changes from normal oral epithelium to OSCC.

In the OPMDs’ TIME, tumor-associated fibroblast also plays an important role. A previous study showed the gradual increased involvement of these cells with the progression from mild, moderate, and severe dysplasia to OSCC [75] (Figure 3).

## 9. Enhancing Tumor Treatment Through Immune Modulation and Combination Therapies

Head and neck cancer treatment usually involves multiple approaches. For oral cavity cancer, surgery is typically followed by chemoradiotherapy (CRT). In cases where comorbidities restrict the use of standard cytotoxic chemotherapy, the EGFR monoclonal antibody cetuximab is often combined with radiation therapy. Beyond conventional treatments, PD-L1 and immune checkpoint inhibitors represent advanced approaches in the management of OSCC. A high level of PD-L1 expression within a tumor contributes to immune evasion, fostering resistance to treatment. This overexpression is also associated with increased tumor recurrence and reduced disease-specific survival in OSCC [76]. The FDA has approved the immune checkpoint inhibitors pembrolizumab and nivolumab for the treatment of recurrent or metastatic HNSCC. Both pembrolizumab and nivolumab target the PD-1 receptor on T cells, blocking its interaction with PD-L1 and PD-L2 on tumor cells, thereby enhancing immune-mediated tumor destruction. Understanding PD-1/PD-L1 levels is crucial when combining chemotherapy with immunotherapy, as these biomarkers influence treatment efficacy and guide therapeutic strategies. The effectiveness of PD-L1 inhibitors in OSCC may be limited, particularly in cases where a high PD-L1 expression originates directly from cancer cells rather than immune cells [77]. This finding underscores the need for more personalized treatment approaches, potentially integrating PD-L1 inhibitors with additional therapies to enhance outcomes for patients with locally advanced OSCC.

Recently, a study showed that cisplatin, a chemotherapy drug commonly used in the treatment of OSCC, upregulates PD-L1 and as a result, potentially limits cytotoxic T cell activity [78]. However, curcumin, a well-established anti-cancer and chemo-preventive property and a phytochemical drug, downregulated PD-L1 and reduced regulatory T cell populations [79]. Meanwhile, radiotherapy combined with immune checkpoint inhibition induces PD-L1 expression on immune cells and carcer-associated fibroblast, enhancing the effects of checkpoint inhibitors [80]. Ferroptosis is a newly recognized mechanism of regulated cell death. A high FP score correlated with a low gene copy number burden and a high immune checkpoint expression, suggesting that targeting ferroptosis pathways could enhance immunotherapy and chemotherapy responses in OSCC [81].

There are some promising breakthrough methods being developed in OSCC treatment. Zhang XK et al. in 2023 developed a biohybrid system based on *Spirulina platensis* (SP) and the STING agonist ADU-S100 [82]. This system enhanced immune cell infiltration and alleviated hypoxia in the OSCC TME, which improved the effectiveness of PD-1 therapy. Their findings suggest a novel approach for enhancing immunotherapy outcomes by targeting the TME. Tumor-associated macrophages (TAMs) are key players in the tumor microenvironment and significantly influence treatment response. Research explored the immunosuppressive effects of PLIN2 (adipose differentiation-related protein) in OSCC, specifically TAMs. PLIN2 belongs to the perilipin family and is a marker of lipid droplets. High levels of PLIN2 in TAMs correlated with immune suppression and a poorer prognosis, suggesting that targeting PLIN2 could help restore immune balance in the TME [83].

Kondo Y et al. investigated in 2021 the immunosuppressive role of TGF-beta in OSCC. They found that TGF-beta inhibits the cytotoxic T lymphocyte (CTL) function by promoting the expansion of Tregs and cancer-associated fibroblasts. Their work supports the use of TGF-beta inhibitors in combination with immune checkpoint inhibitors to restore CTL function in OSCC [84].

Emerging therapeutic strategies and combination therapies are showing great promise in enhancing cancer treatment outcomes. Su W et al. proposed a novel approach that integrates TME-reactive oxygen species/pH dual-responsive nano prodrugs and oncolytic herpes simplex virus (HSV-1) therapy [85]. This combination effectively reshapes the tumor microenvironment, boosting immune response and transforming “cold” tumors into “hot” ones, thereby enhancing the efficacy of immune checkpoint inhibitors. Similarly, Wu C et al. explored a new combination therapy in a Phase II clinical trial for esophageal squamous cell carcinoma (ESCC), evaluating a neoadjuvant regimen of PD-1 blockade, nanoparticle albumin-bound paclitaxel (nab-paclitaxel), and S-1. Their study provided valuable insights into drug resistance mechanisms and demonstrated the potential of spatial proteomics to guide personalized cancer treatment, highlighting the promise of these emerging therapeutic approaches [86].

Integrating photodynamic and photothermal therapies with immune modulation in oral cancer is showing promising outcomes by not only directly destroying cancer cells but also enhancing immune response. These approaches utilize advanced nanomaterials and photosensitizers to generate reactive oxygen species and heat upon light exposure, effectively killing tumor cells. This process induces immunogenic cell death, releasing tumor antigens and stimulating the immune system, which boost the infiltration of cytotoxic T cells into the tumor microenvironment. Here are some important breakthroughs that have significantly advanced the treatment: Shi et al., 2023, developed a bacterial nanomedicine from *Porphyromonas gingivalis* (nmPg) using protoporphyrin IX as a photosensitizer to enhance PDT and activate immune responses against OSCC [87]. Adding doxorubicin (DOX) showed synergistic effects, significantly inhibiting tumor growth and metastasis. Meanwhile, Zhou JY et al. developed in 2022 the nanocomposite TiO2@Ru@siRNA, combining a ruthenium-based photosensitizer with siRNA targeting HIF-1α. It triggers dual photodynamic effects, damaging cancer cells and reducing hypoxia while activating T cells and lowering immunosuppressive factors, leading to significant tumor inhibition in animal models [88]. Zhang L et al. in 2023 used graphdiyne oxide in PDT to increase the stiffness of OSCC cells, making them more vulnerable to CD8+ T cells [89]. Also, Liu JC et al. developed in 2024 the carrier-free nanocomplex NPsR, combining Chlorin e6 (Ce6) and the BRD4 inhibitor JQ1. This combination induces immunogenic cell death, reduces PD-L1 expression, and enhances immune response. The addition of the RGD peptide facilitates targeted delivery, boosting PDT efficacy and overcoming resistance [90].

These studies highlight the potential of integrating photodynamic therapy (PDT) and photothermal therapy (PTT) with immune modulation, chemotherapy, and advanced drug delivery systems for OSCC treatment. By addressing tumor heterogeneity, invasiveness, and immune evasion, these strategies offer promising avenues to enhance therapeutic efficacy, reduce recurrence, and improve overall patient outcomes in managing OSCC.

## 10. Conclusions

One of the critical challenges in understanding OPMD is the oral cavity’s unique environment that combines well-developed innate and adaptive immune responses. In this context, oral keratinocytes interact with immune cells and mediators linked to the pathogenesis of cancer, including increased rates of the malignant transformation of OPMDs. The literature shows overwhelming data to support that the immune microenvironment has a direct role in promoting invasion and the malignant transformation of oral keratinocytes. This makes OSCC a unique cancer that arises in a typically inflamed environment and that hijacks that same immune response to progress and invade.

The mechanisms by which oral cancer pathogenesis and progression are influenced by inflammation are complex and involve inflammatory mediators, immune cells, and other stromal cells in the TIME. Further research is needed to translate these novel findings into new diagnostic, prognostic, and therapeutic options for OSCC.

## Data Availability

Not applicable.

## Abbreviations

4NQO	4-Nitroquinoline 1-oxide
ADU-S100	STING Pathway Agonist
AP2M1	Adaptor related protein complex 2 mu 1 subunit
Arg1	Arginase 1
BRD4	Bromodomain Containing Protein 4
CAFs	Cancer-Associated Fibroblasts
Cal-27	Human oral squamous cell carcinoma (OSCC) cell line.
CATSPER1	Cation channel sperm-associated 1
CCL2/CCR2	Chemokine (C-C Motif) Ligand 2 / Receptor 2
CCL9/CCR1	Chemokine (C-C Motif) Ligand 9 / Receptor 1
CCR7	CC Chemokine Receptor 7
CD4+	Cluster of Differentiation 4 Positive
CD45RO+	Memory T Cell Marker
CD57+	Natural Killer Cell Marker
CD8+	Cluster of Differentiation 8 Positive
Ce6	Chlorin e6
CMTM6	CKLF-like MARVEL Transmembrane Domain-containing 6.
CRT	Chemoradiotherapy
CTLs	Cytotoxic T Lymphocytes
CXCL14	C-X-C Motif Chemokine Ligand 14
CXCR1/2/4	C-X-C Chemokine Receptor 1/2/4
DCs	Dendritic Cells
DOX	Doxorubicin
EGFR	Epidermal Growth Factor Receptor
ErbB2	Erythroblastic Leukemia Viral Oncogene Homolog 2
ERK1/2	Extracellular Signal-Regulated Kinases 1 and 2
ESCC	esophageal squamous cell carcinoma
FDA	Food and Drug Administration
FISH	Fluorescence In Situ Hybridization.
Foxp3	Forkhead box P3
FP score	Ferroptosis Score
GM-CSF	Granulocyte-Macrophage Colony-Stimulating Factor
HFD	high-fat diet
HIF-1α	Hypoxia-Inducible Factor 1-alpha
HNSCC	Head and Neck Squamous Cell Carcinoma
HSP90B1	Heat shock protein 90 beta family member 1
HSV-1	Herpes Simplex Virus Type 1
IFN-β	Interferon beta
IFN-γ	Interferon-gamma
IL-23/IL-12/IL-10/IL-6/IL-1	Interleukin-23/12/10/6/1
iNOS	inducible Nitric Oxide Synthase
JAK	Janus Kinase
JQ1	BET Bromodomain Inhibitor
KIT	KIT proto-oncogene, receptor tyrosine kinase
LAT	Linker for activation of T cells
LINC00996	Long intergenic non-protein coding RNA 996
M-MDSCs	monocytes- derived Myeloid-Derived Suppressor Cells
M1	anti-tumor macrophages
M2	pro-tumor macrophages
MCP1	monocyte chemotactic protein-1
MCs	Mast Cells
MDSCs	Myeloid-Derived Suppressor Cells
MHC I	Major Histocompatibility Complex Class I
MHC II	Major Histocompatibility Complex Class II
MIP-1α	macrophage inflammatory protein-1alpha
MMPs	Matrix Metalloproteinases
N1	anti-tumor neutrophils
N2	pro-tumor neutrophils
nab-paclitaxel	Nanoparticle Albumin-Bound Paclitaxel
NETs	Neutrophil Extracellular Traps
NLR	Neutrophil-to-Lymphocyte Ratio
nmPg	Nanomedicine from Porphyromonas gingivalis
NOS	oxide synthase
NPsR	Nanoparticles loaded with Rose Bengal
OLE	Oral Lupus Erythematosus
OLP	Oral lichen planus
OPMDs	Oral Potentially Malignant Disorders
OSCC	Oral Squamous Cell Carcinoma
OSF	Oral Submucous Fibrosis
PAMPs	Pathogen-Associated Molecular Patterns.
PD-1	Programmed Cell Death Protein 1
PD-L1	Programmed Death-Ligand 1
PD-L2	Programmed Death-Ligand 2
PDT	Photodynamic Therapy
PI3K/AKT	Phosphoinositide 3-Kinase / Protein Kinase B
PLIN2	adipose differentiation-related protein
PMN-MDSCs	Polymorphonuclear Myeloid-Derived Suppressor Cells
PTT	Photothermal Therapy
PVL	Proliferative Verrucous Leukoplakia
RAB32	RAB32, member RAS oncogene family
RANTES	Regulated upon Activation, Normal T cell Expressed and Secreted
RGD peptide	Arginine-Glycine-Aspartic Acid Peptide
RNS	Reactive Nitrogen Species
ROS	Reactive Oxygen Species
S-1	Oral Fluoropyrimidine Anti-Cancer Drug
SCC25	human oral squamous cell carcinoma (OSCC) cell line
SMYD3	ET and MYND domain containing 3
SOCS1	Suppressor of cytokine signaling 1
SP	Spirulina platensis
STAT3, 6	Signal Transducer and Activator of Transcription 3, 6
SX-682	a small-molecule dual inhibitor of the chemokine receptors CXCR1 and CXCR2
TAMs	Tumor-Associated Macrophages
TANs	Tumor-Associated Neutrophils
TGF-β	Transforming Growth Factor Beta
TH2/17	T helper type 2/17 cells
THBS1	Thrombospondin 1
TIGIT	T cell immunoreceptor with Ig and ITIM domains
TILs	Tumor-Infiltrating Lymphocytes
TIME	Tumor Immune Microenvironment
TiO_2_@Ru@siRNA	Titanium Dioxide-Ruthenium Conjugated Small Interfering RNA
TNF-α	Tumor Necrosis Factor Alpha
Tregs	Regulatory T Cells
TβRII	Transforming Growth Factor Beta Receptor Type II
VEGF	Vascular Endothelial Growth Factor
